# CRIP1 expression is correlated with a favorable outcome and less metastases in osteosarcoma patients

**DOI:** 10.18632/oncotarget.398

**Published:** 2011-12-24

**Authors:** Daniel Baumhoer, Mareike Elsner, Jan Smida, Stephanie Zillmer, Sandra Rauser, Cédrik Schoene, Benjamin Balluff, Stefan Bielack, Gernot Jundt, Axel Walch, Michaela Nathrath

**Affiliations:** ^1^ Bone Tumor Reference Center at the Institute of Pathology, University Hospital Basel, Basel, Switzerland; ^2^ Clinical Cooperation Group Osteosarcoma, Helmholtz Zentrum Muenchen - National Research Centre for Environment and Health, Neuherberg, Germany; ^3^ Institute of Pathology, Helmholtz Zentrum Muenchen - National Research Centre for Environment and Health, Neuherberg, Germany; ^4^ Department of Pediatrics, Technische Universitaet Muenchen and Pediatric Oncology Center, Munich, Germany; ^5^ Klinikum Stuttgart Olgahospital, Pediatrics 5 - Oncology, Hematology, Immunology, Stuttgart, Germany

**Keywords:** Osteosarcoma, cysteine-rich intestinal protein 1, CRIP1, prognosis, metastases

## Abstract

Predicting the clinical course of osteosarcoma patients is a crucial prerequisite for a better treatment stratification in these highly aggressive neoplasms of bone. In search of new and reliable biomarkers we recently identified cysteine-rich intestinal protein 1 (CRIP1) to have significant prognostic impact in gastric cancer and therefore decided to investigate its role also in osteosarcoma. For this purpose we analyzed 223 pretherapeutic and well characterized osteosarcoma samples for their immunohistochemical expression of CRIP1 and correlated our findings with clinico-pathological parameters including follow-up, systemic spread and response to chemotherapy. Interestingly and contrarily to gastric cancer, we found CRIP1 expression more frequently in patients with long-term survival (10-year survival 73% in positive vs. 54% in negative cases, p = 0.0433) and without metastases (p = 0.0108) indicating a favorable prognostic effect. CRIP1 therefore seems to represent a promising new biomarker in osteosarcoma patients which should be considered for a prospective validation.

## INTRODUCTION

Osteosarcomas are the most common primary malignant tumors of bone generally following an aggressive clinical course [1]. The high rate of systemic spread already at the time of diagnosis explains the efficacy of neoadjuvant and adjuvant chemotherapy and the dismal prognosis after radical surgery alone. However, although 5-year survival rates of up to 50-70% can be achieved using current treatment protocols, a substantial group of patients with metastatic, recurrent and/or refractory disease is still left without effective treatment options [2, 3]. Analyzing the response to chemotherapy histologically or screening for a set of distinct chromosomal aberrations as we proposed only recently can help in predicting the prognosis of osteosarcoma patients but does not allow a sufficient risk assessment for further treatment stratification [4, 5]. Identifying patients who may not respond to first-line chemotherapy or will have an increased likelihood of developing metastases therefore seems to be a crucial precondition for differentiating high and low risk patients and for designing more individualized therapy regimens. Consequently, appropriate and reliant biomarkers are urgently needed.

Cysteine-rich intestinal protein 1 (CRIP1) is a member of the LIM family of zinc-finger proteins which are thought to be involved in cellular growth and differentiation [6, 7]. In several studies, CRIP1 has been proposed as a novel biomarker for breast cancer and its precursor lesions which can be triggered by ERBB2 overexpression [8-10]. Subsequently, upregulation of CRIP1 was also detected in colorectal, cervical and prostatic cancer whereas downregulation was demonstrated in pancreatic carcinoma [11-15]. In gastric cancer, however, we were only recently and for the first time able to demonstrate a pivotal prognostic impact for CRIP1. Overexpression resulted in a significantly shorter overall survival and was identified to represent the strongest prognostic variable besides nodal status [16]. Since the role of CRIP1 in osteosarcomas has not been studied so far, we investigated a set of 223 pretherapeutic tumor samples immunohistochemically and correlated our findings with clinico-pathological parameters to also identify potential prognostic implications of CRIP1 in these aggressive tumors of bone.

## RESULTS

### Immunohistochemical expression of CRIP1

**Figure 1:**
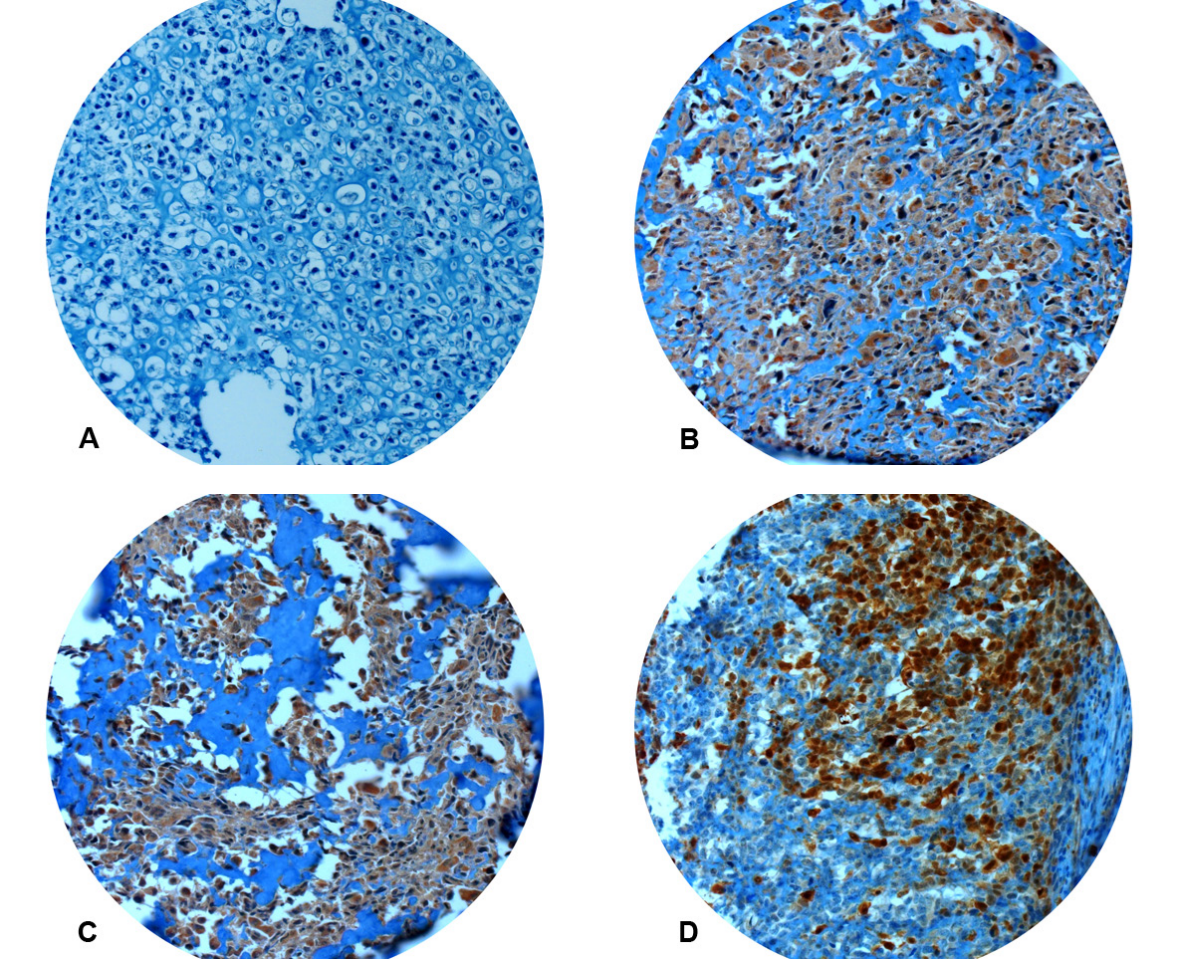
Immunhistochemistry for CRIP1 Absent (A) or focal (< 50% positive tumor cells) immunoreactivity was considered negative. Strong and constant staining (B, C) or immunoreactivity in more than 50% of tumor cells (D) was regarded CRIP1 positive. All pictures x200.

All but four cases demonstrated strong and consistent immunoreactivity for vimentin. Those four cases were excluded from the evaluation leaving a total of 219 osteosarcoma cases for further analysis. CRIP1 expression was considered positive when more than 50% of tumor cells were immunoreactive for the respective protein (Figure 1). In total, CRIP1 was evaluable in 155/219 (71%) and considered positive in 69/155 (45%) cases. Drop out of samples was mainly due to cutting artefacts and/or lack of sufficient amounts of tumor tissue per punch.

### Correlation of CRIP1 expression with clinico-pathological parameters

**Figure 2:**
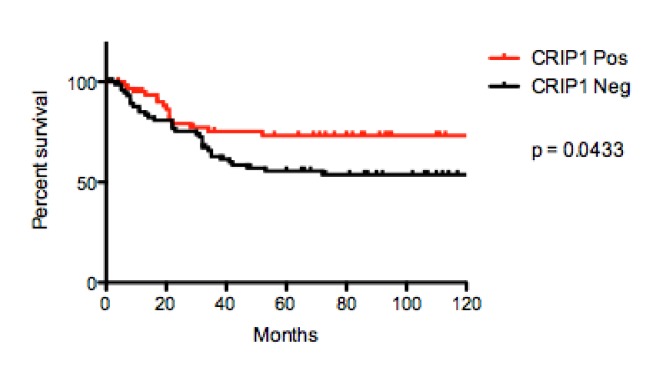
Kaplan-Meier curves comparing 10-year survival of osteosarcomas with and without CRIP1 expression

The 10-year survival rate (10-YSR) differed significantly between CRIP1 positive and negative cases (73% vs. 54%, p = 0.0433, Figure 2). Additionally, CRIP1 positive cases had a significantly lower frequency of systemic spread (p = 0.0108, Table 2). There were no statistically significant correlation between the expression of CRIP1 and the response to chemotherapy (Table 2).

**Table 1:** Patients' characteristics

**Sex**	**208/223 (93%)**^1^
male	106
female	102
**Age at diagnosis**	210/223 (94%)^1^
average	22.9 years
median	16.3 years
range	4-88 years
**Localisation**	212/223 (95%)^1^
Femur	103
Tibia	50
Humerus	17
Fibula	11
Pelvis	11
other	20
**Metastases (lung)**^2^	202/223 (91%)^1^
yes (total)	89
yes (at initial diagnosis)	24
no	113
Time to metastases average	15.6 months
Time to metastases median	12.4 months
Time to metastases range	0-79 months
**Follow-up**	214/223 (96%)^1^
average	65 months
median	41.7 months
range	0-286.7 months
**Survival**	214/223 (96%)^1^
died	79
alive	135
**Response to chemotherapy**^2^	146/223 (66%)^1^
Good	87
Poor	59

1 X/223 (Y%) = data available in X cases (Y% of the total number of cases)

2 There were no patients included with metastases to other organs that did not also have metastases to the lungs

3 Good response = < 10% vital tumor cells in the resection specimen, poor response = ≥ 10% vital tumor cells

**Table 2:** Correlation between protein expression and metastases / response to chemotherapy

	**p-value**
Metastases and expression of CRIP1	**0.0108**
Response to chemotherapy and expression of CRIP1	0.4808

## DISCUSSION

Osteosarcoma tissue samples are rare, especially since resected specimens usually have undergone neoadjuvant treatment hampering further molecular analyses. Only if the material of the initial biopsy is not completely needed for diagnostic purposes it can be used for experimental studies which, thus, have to be carefully planned to analyze the valuable tissue samples as efficiently as possible. One approach to ensure optimal utilization of osteosarcoma samples is to construct tissue microarrays like the one we used in this study. Since the identification of novel biomarkers seems mandatory for developing better treatment stratifications and for recognizing patients at high risk for systemic spread or chemoresistance, these arrays are an essential tool for the analysis of large numbers of samples under the same technical conditions.

CRIP1 is a zinc-binding protein that was discovered as an intestinal-specific developmentally regulated gene and has subsequently been recognized in several other tissues and cells [6, 17]. In the last years, deregulated CRIP1 expression was reported to characteristically occur in several malignant tumors, including breast, cervical, prostatic, colorectal, and pancreatic cancer [9, 11, 12, 14, 15]. Interestingly, CRIP1 was also considered a bone-specific breast cancer metastasis gene [18, 19]. Although its precise function is still to be elucidated, CRIP1 seems to influence various cellular and immunological signalling pathways involved in cellular differentiation, protein-protein interactions during transcription, immune response, and cytokine expression [6, 7]. Additionally, a role in ERBB2-related oncogenesis was proposed for CRIP1 since upregulation of CRIP1 was recorded in ERBB2-overexpressing carcinomas of the breast which generally follow an aggressive course [10]. Although this already indicated an indirect prognostic effect for CRIP1, we demonstrated only recently and for the first time a crucial prognostic impact for CRIP1 expression itself. In gastric cancer, immunohistochemical detection of CRIP1 was associated with a significantly shorter overall survival and represented the most decisive prognostic factor besides lymph node status [16]. In the present study, however, we identified CRIP1 to be strongly associated with a favorable outcome and as a statistically significant negative predictor of systemic spread in osteosarcoma. Our findings thus indicate CRIP1 to have tumor type specific oncogenic and tumor suppressor properties needing further pathogenetic clarification. Nevertheless, osteosarcomas are not the first malignant tumors in which a tumor suppressor function of CRIP1 is highly suggestive since also in carcinomas of the pancreas a downregulation of CRIP1 has been reported [14]. However, evidence of prognostic impact of CRIP1 in pancreatic cancer is still lacking.

Taken together, we identified CRIP1 as a new and promising biomarker in osteosarcoma. Although a significant correlation with long-term survival and metastatic spread was identified, an association with response to chemotherapy was not. This phenomenon might be influenced by a relatively high drop out of cases due to technical issues and available response data in only 66% of cases. Consequently, CRIP1 should be validated prospectively to proof its prognostic reliability and, nonetheless, a potential role in response prediction in osteosarcoma patients.

## METHODS

### Tissue samples and patients' characteristics

All tissue samples were obtained from the archives of the Bone Tumor Reference Center at the University Hospital Basel and the Clinical Cooperation Group Osteosarcoma at the Helmholtz Zentrum Muenchen and comprised cases that were diagnosed between 1974 and 2010. Only samples from patients without prior treatment were included in the study (n = 223). Full patients' characteristics are presented in Table 1.

### Immunohistochemistry

Tissue samples (n = 223) were fixed in buffered 4% formalin, decalcified using EDTA (ethylenediaminetetraacetic acid) if required, embedded in paraffin, and used to construct tissue microarrays as described elsewhere [20]. The resulting blocks were cut into 3 μm sections that were transferred to glass slides and subsequently used for immunohistochemistry. To ensure proper immunoreactivity of tumor samples, immunohistochemistry for vimentin was performed according to routine protocols (Ventana BenchMark XT®, Roche, Basel, Switzerland; CC1 pretreatment; prediluted antibody, clone V9; DAB chromogen). Immunohistochemistry for CRIP1 was also carried out using an automated stainer (Ventana DISCOVERY XT®, Roche, Basel, Switzerland; DAB chromogen). The dilution used for the primary antibody against CRIP1 (AbD Serotec, Oxford, UK) was 1:100.

### Evaluation of immunohistochemistry against CRIP1

Immunoreactivity for CRIP1 was scored semi-quantitatively by evaluating the number of positive cells over the total number of cells. Both cytoplasmic and nuclear staining was evaluated. In cases with more than one punch per tumor the average expression was determined for further analyses. Punches that were not completely enclosed on the sections or showed artefacts due to sectioning were excluded from the analysis.

### Statistical analyses

In order to determine the cut-off scores defining immunohistochemical CRIP1 positivity and negativity, receiver operating characteristic (ROC) curve analysis was performed with the endopoint of survival [21]. Survival analyses were carried out using the Kaplan-Meier and Log-rank (Mantel-Cox) test. The differences in protein expression between patients with and without metastases and with good and poor response to chemotherapy were determined using the Mann-Whitney-Test. Only p-values < 0.05 were considered statistically significant. All analyses were conducted using GraphPad Prism 5.0d (La Jolla, CA, USA) and SPSS 19 (IBM Corporation, Armonk, NY, USA).
